# Inferring Processes from Spatial Patterns: The Role of Directional and Non–Directional Forces in Shaping Fish Larvae Distribution in a Freshwater Lake System

**DOI:** 10.1371/journal.pone.0050239

**Published:** 2012-11-20

**Authors:** Andrea Bertolo, F. Guillaume Blanchet, Pierre Magnan, Philippe Brodeur, Marc Mingelbier, Pierre Legendre

**Affiliations:** 1 Interuniversity Research Group in Limnology and Aquatic Environment (GRIL), Québec, Canada; 2 Research Centre for Watershed - Aquatic Ecosystem Interactions (RIVE) - Université du Québec à Trois-Rivières, Trois-Rivières, Québec, Canada; 3 Département de Sciences Biologiques, Université de Montréal, Montréal, Québec, Canada; 4 Department of Renewable Resources, University of Alberta, Edmonton, Alberta, Canada; 5 Section of Ecology, Department of Biology, University of Turku, Turku, Finland; 6 Ministère des Ressources Naturelles et de la Faune - Direction de l’expertise de la Mauricie et du Centre-du-Québec, Trois-Rivières, Québec, Canada; 7 Ministère des Ressources Naturelles et de la Faune - Service de la Faune Aquatique, Québec, Canada; Institute of Marine Research, Norway

## Abstract

Larval dispersal is a crucial factor for fish recruitment. For fishes with relatively small-bodied larvae, drift has the potential to play a more important role than active habitat selection in determining larval dispersal; therefore, we expect small-bodied fish larvae to be poorly associated with habitat characteristics. To test this hypothesis, we used as model yellow perch (*Perca flavescens*), whose larvae are among the smallest among freshwater temperate fishes. Thus, we analysed the habitat association of yellow perch larvae at multiple spatial scales in a large shallow fluvial lake by explicitly modelling directional (e.g. due to water currents) and non-directional (e.g. due to aggregation) spatial patterns. This allowed us to indirectly assess the relative roles of drift (directional process) and potential habitat choice on larval dispersal. Our results give weak support to the drift hypothesis, whereas yellow perch show a strong habitat association at unexpectedly small sizes, when compared to other systems. We found consistent non-directional patterns in larvae distributions at both broad and medium spatial scales but only few significant directional components. The environmental variables alone (e.g. vegetation) generally explained a significant and biologically relevant fraction of the variation in fish larvae distribution data. These results suggest that (i) drift plays a minor role in this shallow system, (ii) larvae display spatial patterns that only partially covary with environmental variables, and (iii) larvae are associated to specific habitats. By suggesting that habitat association potentially includes an active choice component for yellow perch larvae, our results shed new light on the ecology of freshwater fish larvae and should help in building more realistic recruitment models.

## Introduction

Larval dispersal is crucial to several fish species, determining, among other things, the chances of individuals to settle in optimal habitats or the level of connectivity among populations [1]. Many fishes begin their pelagic larval stage as plankton but end it as nekton, gradually improving their ability to influence their dispersal [2]. A better knowledge of the ontogeny of this transition will improve our understanding of dispersal patterns and, in turn, our ability to integrate behavior into dispersal models. It is now recognized, at least for marine species, that even small larvae can select the habitat in which they settle [2], but there is limited knowledge on the relative roles of drift versus habitat selection in shaping the spatial distribution of fish larvae. Because most larval stages of fishes have limited locomotion capabilities, processes governing their distribution in large systems should act across multiple spatial scales; passive processes should dominate at broad scales, while active habitat selection should dominate at smaller scales.

Among temperate freshwater fish species, yellow perch (*Perca flavescens*) is probably one of the few showing a clear larval pelagic phase with a high potential to drift offshore over long distances [3], [4]. Like for the ecologically similar Eurasian perch (*Perca fluviatilis*), the duration of the larval phase in yellow perch is relatively long: the pelagic stage of *Perca* spp. begins shortly after larvae hatch in the littoral zone and lasts 2–3 weeks in small lakes to approximately 40 days in larger lakes [5], [6]. The duration of the pelagic period may be even longer in systems driven by marine-like hydrodynamics such as the Great Lakes, with some individuals captured in the pelagic zone after 75 days [3], [4]. This extended pelagic phase suggests that spatially and temporally variable current patterns could be crucial for yellow perch larvae timing of settlement (e.g. [4]). Whereas *Perca* spp. habitat shifts were typically studied in lacustrine systems with a narrow littoral zone and a well-defined pelagic habitat [6], [7], we have poor knowledge about this phenomenon in systems lacking a true offshore habitat.

In this study, we modelled the spatial distribution of yellow perch larvae collected in Lake St. Pierre (LSP), the largest fluvial lake of the St. Lawrence River, which is the remnant of former postglacial Lake Lampsilis. LSP is dominated by shallows with patchy vegetation, excepted for the exclusion of the main channel, and is considered an important fish nursery area [8]. LSP is thus an ideal system to analyze early habitat associations in yellow perch in systems lacking a true pelagic zone. Its heterogeneous hydrodynamics along the transversal axis makes it particularly interesting since LSP could be seen as a large river with fast directional flow in the main channel and extensive slow-flowing zones with limited transversal exchange between adjacent water masses [9], [10]. These characteristics could potentially lead to larval drift in some areas and retention in other areas with slower current [11], [12].

Preliminary results from field sampling indicated that, at the peak of their pelagic phase, the highest abundances of larvae are found at the interface of these two habitats, in water masses where currents are close to the swimming speed of the newly hatched yellow perch (1 cm sec^−1^) [13]. However, since velocity measurements are not available for the years sampled in this study and discharge differs among years, it is rather difficult to predict the importance of drift on yellow perch larvae distribution in LSP. To overcome these limitations, we adopted a method based on multiple *a priori* hypotheses to infer processes from spatial patterns [14]. Following this approach, we tested hypotheses about the relative importance of directional (e.g. due to water currents) and non-directional processes (e.g. due to aggregation), used as proxies of drift and potential active habitat choice, respectively, on yellow perch larvae distribution at multiple spatial scales.

We structured our modeling approach in two main steps. In a first step, we partitioned the variation of the abundance data [15] to model the spatial patterns of yellow perch larvae under hypotheses of presence (asymmetric eigenvector maps – AEM) or absence (Moran’s eigenvector maps – MEM) of directional processes [16]–[18]. This approach allowed us to compare the relative ability of AEM and MEM to model larval abundance and therefore to infer about the relative importance of directional and non-directional processes in shaping their distribution. By explicitly testing hypotheses related to the role of directional vs. non-directional processes on abundance of yellow perch larvae at different scales, we wanted to highlight the importance of drift and potential habitat choice as potential determinants of larval distribution. In a second step, variation partitioning at different spatial scales also allowed the assessment of the independent contributions of spatial and environmental variables (e.g. abundance of aquatic vegetation) on larvae abundance to determine the potential of the larvae to select their habitat. Our two-steps approach thus allowed us to asses the relative roles of drift and potential active habitat choice in determining the distribution of yellow perch larvae at multiple scales, which were defined on the basis of the autocorrelation properties of the spatial models built to analyze the larval abundance data.

**Figure 1:**
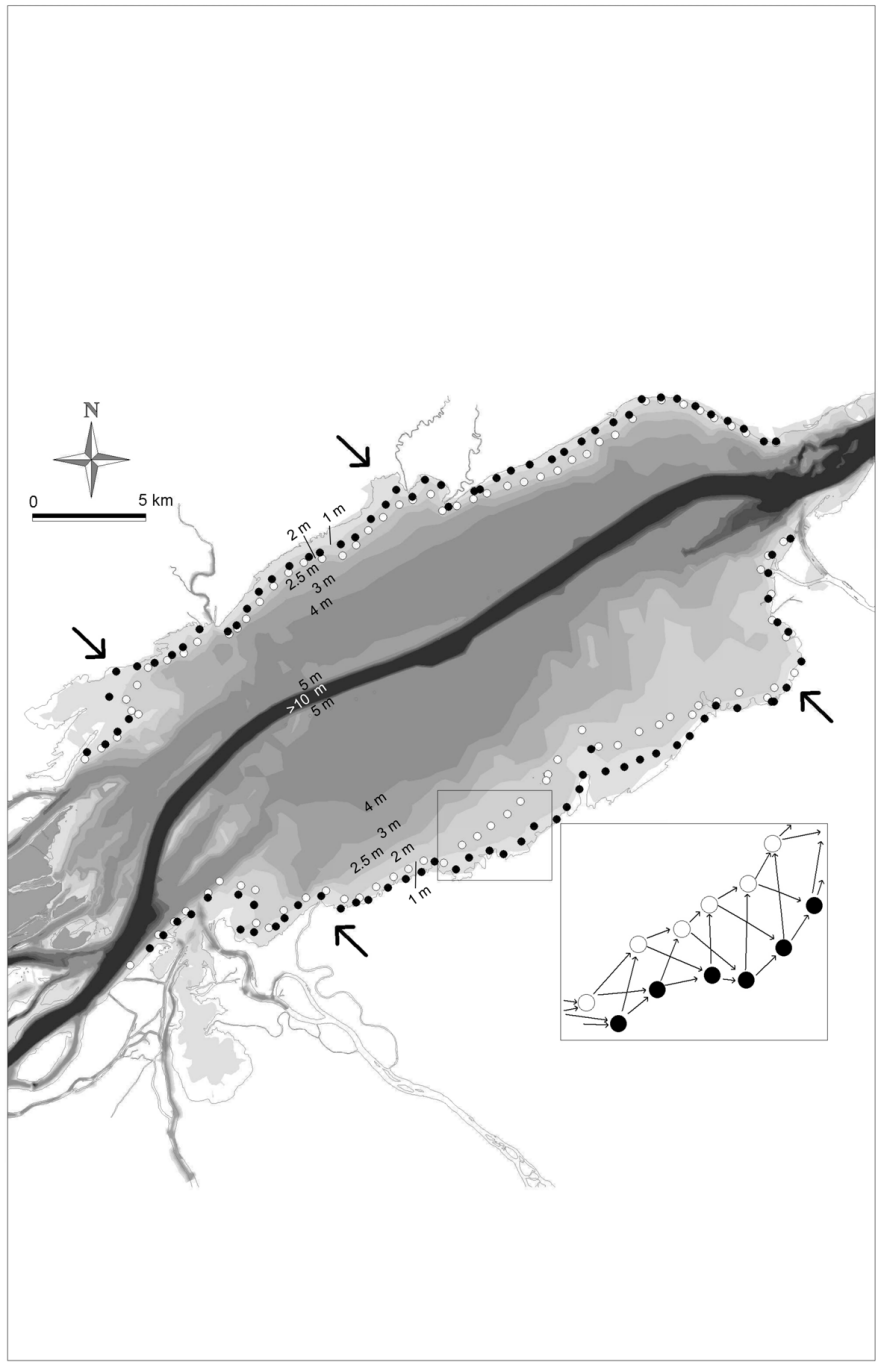
Study system and sampling sites. Lake St. Pierre, St. Lawrence River, Québec, Canada. Filled and open circles represent the locations of the sampling sites in 2005 at the 60–80 cm isobath and at the 80–120 cm isobath, respectively. The inset shows for a subset of sampling sites the general structure of the connection diagram used to build the AEM eigenfunctions. In 2006 and 2007, the sampling sites were located close to the 2005 sites, but adjusted for the water level so that they were located at the pre-determined isobaths. The connection diagram was in some cases adapted to take account of missing data. Arrows indicate the approximate locations of the main known spawning grounds. The main channel of the St. Lawrence River (>10 m deep; dark grey) flows from S-W to N-E. Depth contours are based on averaged water levels in June.

Based on our knowledge of yellow perch larval ecology in our study system, we made the following predictions: (i) at broad scale (ca. 4.5 to 30 km) (i.e. at the between-spawning area scale; Fig. 1), yellow perch distribution patterns should be mainly non-directional because they are controlled by static factors such as the locations of the main spawning areas and should have similar importance from year to year. Yellow perch larvae were expected to show large scale aggregation patterns in correspondence with the spawning areas but, because of relatively weak currents in the near-shore study areas and the short time since hatching, directional processes along the river longitudinal axis should not be important at that scale (MEM should thus outperform or perform equally well with AEM at that scale); (ii) at medium (ca. 3 to 4.5 km) and fine scales (ca. 2 to 3 km) (i.e. at the within-spawning area scale), the spatial patterns should be caused by downstream flow (i.e. AEM should outperform MEM at both scales); and (iii) while controlling for the spatial components, no relationship was expected locally (i.e. at the scale of the sites) between larvae abundance and the environmental variables, since we assumed that larval dispersal was mainly passive and driven both by directional (e.g. drift) and/or non-directional hydrodynamic factors (e.g. wave action at spawning locations).

## Methods

### Study Area

Lake St. Pierre (LSP; 46°12′N; 72°50′W) is the largest (315 km^2^) fluvial lake of the St. Lawrence River (Québec, Canada). The north and south shore areas of the lake are shallow (<3 m) and separated by a deep (>10 m) navigation channel (Fig. 1). LSP is characterized by distinct water masses forming a series of lanes parallel to the main channel and extending the length of the entire system (see Fig. 1 in [10]). The floodplain of LSP, covering approximately 14 000 ha during 5–9 weeks of the spring freshet, is an extremely important spawning ground for yellow perch. The submerged vegetation is dominated by *Vallisneria americana*, *Potamogeton richardsonii*, and *P*. *pectinata*. The emergent vegetation is patchy and dominated by *Schoenoplectus lacustris*, *Sagittaria latifolia* and *Sparganium eurycarpum*. The fish community is composed of up to 80 species [19].

### Larvae Sampling

Yellow perch larvae were collected during the spring of 2005 (28-May to 10-June), 2006 (05-June to 16-June) and 2007 (28-May to 14-June). Sampling was scheduled to occur approximatively 6–7 weeks after peak spawning of yellow perch (20-April in 2005, 13-April in 2006, and 24-April in 2007; Yves Mailhot, Ministère des Ressources Naturelles et de la Faune du Québec, comm. pers.). A total of 198 sites were sampled once each year during daytime. The sampling sites were located all over the lake perimeter at regular intervals (ca. 700 m) on two isobaths (60–80 cm and 100–120 cm; Fig. 1). Whereas the distance between two contiguous sites along the same isobaths was relatively constant, the distance between two contiguous sites located on different isobaths varied depending on the slope of the bottom between the isobaths (Fig. 1). These isobaths were selected following a pilot study which showed that the abundance of yellow perch larvae was close to zero outside the 50–150 cm depth range during the study period (P. Brodeur, P. Magnan and M. Mingelbier, unpublished data). Our sampling protocol can be considered as a snapshot of the distribution pattern of larvae during the abundance maximum in the pelagic zone, at isobaths where they are potentially subjected to drift. Given the peculiar hydrology of our study system, with lateral water masses slowly flowing along the longitudinal axis and showing little transversal mixing [9], [10], we considered the longitudinal axis as the more interesting to study potential larval drift. Therefore, by analysing the patterns of longitudinal distribution of larvae over different years, our study aimed explicitly at analysing the longitudinal component of larval dispersion in LSP. Because of variations in water levels, the coordinates of the sampling sites were adjusted after 2005 in order to meet the pre-established isobaths: once the exact location was found, we measured the depth of the water column and moved perpendicular to the shore until the exact isobaths was found. Larvae were sampled using push nets at a velocity of 1 m•s–1 (see [20]). Push nets size (a 2 m long plankton-type net, 0.40×0.40 m square mount, 500 µm mesh) was chosen to efficiently sample in shallow waters. A 50-m long transect parallel to the shore was sampled at each site. Larvae were sampled in the top 40 cm at the 60–80 cm isobaths and in the top 80 cm at the 100–120 cm isobaths. Avoiding bottom layers is necessary to safely operate push-nets. After capture, larvae were narcotized with Tricaine methanesulfonate (MS-222™ – Sandoz) and immediately preserved in 75% ethanol for further laboratory analyses. In the laboratory, larvae were individually identified and measured to the nearest 0.1 mm under a binocular microscope. Abundance data were expressed per unit surface (ind m–2) rather than per volume (ind m–3) to standardize data between isobaths.

### Environmental Variables

At each sampling site, we estimated the substrate type as sand, clay, silt or debris. Submerged and emerged aquatic vegetation density was visually evaluated independently using a semi-quantitative scale: 0 (open water), 1 (sparse), 2 (dense, bottom visible), 3 (very dense, bottom not visible but open water at the surface) or 4 (extremely dense, bottom not visible, no open water at the surface). The dominant structure of emerged and submerged vegetation was noted as linear (e.g. *Juncus* sp.), floating (e.g. *Nymphea* sp.) or arbustive (e.g. Potamogeton sp.). Water conductivity (±1 µS/cm) was measured in the field with a WTW-P340i conductivity meter whereas nephelometric turbidity (±0.01 NTU) was measured in the laboratory on a water sample collected at each site using a WTW-550 turbidity meter. Since samples were taken at two different isobaths, a binary variable ‘isobaths’ was included in the list of environmental variables. The water level at the sampling day and the day of the year counted from January 1st were also included in the list of environmental variables. In addition to account for the variability in larvae abundance due to differences in time since hatching (day of the year), these two factors may affect larvae capturability through a dilution effect (water level), or because of larvae growth (day of the year). Temperature was not included in the analyses since most of the variation in this variable was related to the day of the year or differences among the years.

### Statistical Analyses

#### Spatial patterns

We used AEM, MEM, and linear trend surface analyses (hereafter TREND) to explicitly model the spatial correlation present in the larval abundance data at multiple scales [15], [18]. Whereas the AEM approach is explicitly designed to model directional patterns (e.g. due to river flow), the MEM and TREND are meant to model non-directional processes (e.g. due to aggregation). AEM and MEM variables result from a spectral decomposition of the spatial relationship among the sites, whereas TREND is simply a regression of larval abundance against the XY geographic coordinates of the sites.

The spatial relationships among the sites are provided by a connection diagram (Fig. 1); this information is used to construct the AEM and MEM spatial variables. The connection diagram, representing all the possible paths that larvae could take to move from one site to an adjacent one, was constructed from prior knowledge of water movements and water masses in the lake based on satellite imagery and two-dimensional hydrodynamic models [9], [10], [21]. With current speeds higher than 30 cm sec-1 during spring [22], the main channel of the St. Lawrence River can be considered as a near-complete barrier for larvae between the north and the south shores of Lake St. Pierre; for this reason, separate connection diagrams were constructed for the northern and southern portions of the lake, which were analysed separately. The directional links (considering the arrows on the links in Fig. 1) were used to construct the AEM eigenfunctions, whereas the same connection diagrams with arrows in both directions, transformed into two binary matrices of presence-absence of links among sites, were used to construct the MEM eigenfunctions. This approach can be easily applied to other systems (e.g. rivers or oceanic streams), given that a proper connection diagram can be drawn from knowledge of directional processes. The connection diagram of 2005 (Fig. 1) was used as template for 2006 and 2007; the only differences were due to slight changes in the geographic coordinates (see Larvae sampling section) and to missing data, which required slight modifications to the connections (not shown). This sampling design allowed us to highlight the variations due to water currents along the longitudinal axis rather than due to an eventual offshore migration of the larvae. The reduced lateral mixing among water masses, typical of LSP (see Fig. 1 in [10]), supports our choice of focusing on the longitudinal axis. This is the rationale behind the exclusion of arrows in the offshore direction (i.e. perpendicular to the shore) in the connection diagrams.

Since we did not know how the larvae were spatially structured over the theoretical connection diagram, we tested different weighting functions to incorporate into the analysis some measure of the difficulty of moving from one site to another. Five weighting functions were applied during the construction of the AEM and MEM eigenfunctions. The weights are functions of the sampling sites; the distances are computed from the XY coordinates of the sites following [18]. The five functions were: (1) no weights (connection present = 0, absent = 1); (2–3) the weights follow a concave-down function of the distances (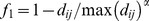); and (4–5) they follow a concave-up function (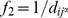); in *f*_1_ and *f*_2_, α is equal to either 1 or 2 [18]. The weights leading to the highest fraction of explained variation in the larval abundance data were retained for further analyses (Online Resource A).

Moran’s *I* coefficients of spatial correlation were computed for each AEM and MEM eigenfunction. They were used to select the eigenfunctions that modelled positive spatial correlation; these had Moran’s *I* values higher than the expected value. The expected value of Moran's *I* is –1/(*n*–1); this is the mean value it would have if calculated for random variables under the normal distribution. Moran’s *I* coefficients were computed for the first-order connections, that is, using only the direct links connecting sites, following [23]; they were tested for significance through 999 random permutations. AEM eigenfunctions were constructed using the “AEM” package [24] in the R statistical language [25], following the procedure presented in Blanchet et al. [18]. MEM eigenfunctions were constructed using the “spacemakeR” package in R [26]. Functions available in these packages were also used to compute the optimum weights for the five weighting functions.

The larval abundance data were modelled by multiple regression via the ***lm()*** function in R by using all eigenfunctions with Moran’s I larger than the expected value (MEM or AEM). Each regression model was recomputed with a canonical redundancy analysis (RDA) using the ***rda()*** function of the “vegan” package [27]. This was done in order to perform permutation tests (999 permutations) with the ***anova.cca()*** function of the “vegan” package; when there is a single response variable, as in the present study, an RDA is simply a multiple regression. This approach was preferred to parametric testing because of the distribution of the larval abundance data. Despite the fact that larvae counts had been log-transformed (*y*′ = log(*y*+1)), we had not eliminated skewness because of the large number of zeros in the count data. Permutation tests of non-normal data, even highly skewed, have correct levels of type I error in multiple regression and are thus valid [28]. Although it is not necessary to remove the trend from the response when selecting AEM eigenfunctions [18], detrended data should be used with MEM eigenfunctions if the TREND analysis is significant [16]. The linear trend was thus removed from the log-transformed response variables (larvae counts) prior to performing MEM analysis by extracting the explained variation due to the XY coordinates of the sampling sites [16].

All analyses were conducted separately at three scales: whereas the TREND modelled patterns only at broad scale, AEM and MEM eigenfuctions were classified as broad, medium or fine-scaled if their Moran’s *I* was >0.75, comprised between 0.75 and 0.45, or <0.45, respectively. This arbitrary choice of the thresholds used to define the scales led to spatial variables corresponding roughly to spatial structures of 4.5–30 km, 3–4.5 km, and 2–3 km in size, respectively. Given that the main bays are 4 to 5 km wide, these three scales are used to model the variability between (broad) or within (medium and fine) the spawning grounds. Within a given type of analysis (i.e. AEM or MEM), spatial eigenfuctions are orthogonal (i.e. linearly independent) by construction, so there is no correlations within or between any of the three groups of spatial variables.

The performance of directional spatial variables in modelling the larval abundance data was compared with that of non-directional ones for each of the three scales separately by variation partitioning [15]. This method uses RDA to partition the variation in species assemblage explained by independent variables into different components (see [15] for computation details). The fractions of variation were calculated from adjusted R^2^ (*R* ^2^_adj_), following Peres-Neto et al. [29], using the ***varpart()*** function of the “vegan” package. This approach allowed an unbiased estimation of the portions of the variation due only to directional spatial processes (AEM), only to non-directional spatial influences (MEM+TREND), or both. For parsimony reasons, the fraction of variation confounded between MEM+TREND and AEM was considered non-directional since that portion of the larval abundance data signal is modeled equally well by directional or non-directional spatial variables. The total non-directional fraction was assumed to be the sum of the “pure” MEM+TREND fraction plus the fraction confounded between MEM+TREND and AEM. In some cases both total directional and non-directional fractions were significantly related to the variation in the abundance of larvae but neither pure fractions were significant (i.e. all the variation was confounded). In these situations, a strict partition of the variation is not possible despite a significant spatial effect, and thus only the non-directional spatial variables were considered in our interpretation. Following this conservative approach, we considered that a directional pattern existed in the larval abundance data only when a significant “pure” AEM fraction was found.

### Habitat Association

The strength of the relationship between larval abundance and habitat features was modelled by using the environmental variables as predictors. Forward procedure was used to select the environmental variables to be included in the regression model following Blanchet et al. [30] through the ***forward.sel()*** function of the “packfor” package [31]. Both the type of substrate and the vegetation are qualitative variables. In order to use them in later analyses, we included them in the analysis as levels of dummy variables following Legendre and Legendre [32]. To avoid creating missing data, the absence of vegetation was coded as a level of a dummy variable as well. A principal component analysis (PCA) was then applied to each set of dummy variables to assure their orthogonality since a different number of observations for each level of a dummy variable might lead to artificial collinearity among the levels. Since no selection was done on the dummy variables, there is no information lost when transforming qualitative variables into quantitative ones using the PCA approach. Each selected axis will subsequently be used as an independent quantitative explanatory variable in the following analyses.

The relative contribution of each environmental variable included in the models was assessed by calculating average semi-partial R^2^ using the “lmg” metric in the “relaimpo” package [33]. Note that the individual contributions may not sum up to the total contribution of the selected environmental variables since these are unlikely to be orthogonal. However, preliminary analyses showed that the collinearity between environmental explanatory variables was not an issue here: the variance inflation factor was always smaller than 10.

Since the environmental variables showed spatial heterogeneity, the variation explained by the environmental and spatial variables may be redundant. To explain this potential redundancy, variations in larvae abundance were analyzed against the spatial and environmental variables combined, using variation partitioning [15]. In this case, TREND will be considered jointly with MEM to represent the non-directional portion of the spatial variation.

All statistical analyses were conducted in the R statistical environment [25].

### Ethics Statement

No specific permits were required for the described field studies since this work was done in collaboration with the Ministère des Ressources naturelles et de la Faune of Québec. The study location is not privately-owned or protected in any way and the field studies did not involve endangered or protected species.

## Results

### Directional vs. Non-directional Patterns

#### 2005 – North shore

A fairly large part of the variation in the larvae abundance data was explained at broad and medium scales (Table 1a). At broad scale, both pure directional and non-directional significant patterns were found, explaining respectively 19.4% and 21.9% of the variation in the larval abundance data. Taking into account the confounded part, non-directional broad spatial patterns accounted for 36.7% of the variation (Table 1a). Therefore, the patterns at this scale are mainly non-directional, but co-occur with a non trivial directional component representing 19.4% of the variation. At medium scale, only non-directional patterns were detected, with an additional 19.7% of the variation in larval abundance data explained at this scale (Table 1a). The mean size of larvae was of 12.1±2.6 mm (n = 924).

**Table 1:** Results of variation partitioning.

a)	North shore	variation partitioning			
Year	Scale	pure directional	Confounded	pure non-directional	total explained
2005	Broad	19.4*	14.8	21.9*	56.1
	Medium	0.0	0.0	19.7*	19.7
	Fine	0.0	0.0	0.0	0.0
2006	Broad	21.2*	18.8	31.3*	71.3
	Medium	12.7*	0.0	0.0	12.7
	Fine	0.0	0.0	0.0	0.0
2007	Broad	0.0	11.9	11.9*	23.8
	Medium	0.0	0.0	0.0	0.0
	Fine	0.0	0.0	9.2*	9.2
**b)**	**South shore**	**variation partitioning**			
**Year**	**Scale**	**pure directional**	**Confounded**	**pure non-directional**	**total explained**
2005	Broad	3.7	63.0	1.4	68.1
	Medium	0.0	0.0	0.0	0.0
	Fine	0.0	0.0	0.0	0.0
2006	Broad	0.0	10.9	0.0	10.9
	Medium	0.0	0.0	0.0	0.0
	Fine	0.0	0.0	0.0	0.0
2007	Broad	0	0	32.2*	32.2
	Medium	9.9*	7.2	8.0*	25.1
	Fine	7.7	4.0	9.4*	21.1

Results of variation partitioning on the log-transformed abundances of yellow perch larvae per square meter in the three study years among three spatial scales. Pure directional components are represented by Asymmetric Eigenvector Maps (AEM), whereas pure non-directional ones are represented by Moran Eigenvector Maps (MEM). The linear trend (TREND) is included in the non-directional component at the broad scale. Fractions are expressed as percentages of the total variation, computed from adjusted coefficient of multiple determination (*R* ^2^_adj_). Asterisks indicate pure fractions that are significant at the 0.05 significance level. Results are presented separately by spatial scale. (a) North shore; (b) South shore.

#### 2005 – South shore

A large portion of the variation was explained at broad scale in the larval abundance data (Table 1b). At this scale, most of the explained variation (63.0%) was confounded between directional and non-directional variables (i.e. explained equally well by both set of variables). Neither the directional, nor the non-directional pure fractions were significant after partitioning (Table 1b). Therefore, the patterns at this scale were considered non-directional with the non-directional spatial variables explaining 64.4% of the variation in the larval abundance data. At medium and fine scales, no significant spatial patterns were detected (Table 1b). The mean size of larvae was of 11.5±2.2 mm (n = 1850).

#### 2006 – North shore

As in 2005, a large portion of the variation was explained at broad and medium scales in the larval abundance data (Table 1a). At broad scale, 18.8% of the variation was confounded between directional and non-directional (MEM and TREND) variables. However, both pure fractions were significant after partitioning and explained relatively large parts of the variation in the larval abundance data (Table 1a). Therefore, the patterns at this scale could be considered as having both a directional and a non- directional component. The non-directional component represented 50.1% of the variation in the larval abundance data, whereas the pure directional fraction was 21.2%. At medium scale, only the directional component was significant and explained 12.7% of the variation in the larval abundance (Table 1a). The mean size of larvae was of 15.6±2.4 mm (n = 594).

#### 2006 – South shore

Variation was only explained at broad scale (10.9%) and was completely confounded between directional and non-directional spatial descriptors (Table 1b). The pattern was thus considered entirely non-directional. The mean size of larvae was of 19.3±2.6 mm (n = 489).

#### 2007 – North shore

Relatively to 2005 and 2006, a smaller fraction of the variation was explained in 2007 at broad scale on the north shore in the larval abundance data (Table 1a). At this scale, nearly half of the explained variation (11.9%) was confounded between directional and non-directional variables. Only the non-directional pure fraction was significant after partitioning (Table 1a). Therefore, the patterns at this scale were considered non-directional; non-directional variables explained 23.8% of the variation in the larval abundance data. The pure directional fraction was negligible. Whereas at medium scale no spatial pattern was detected, we found that at the fine scale 9.2% of the variation was explained by the non-directional spatial variables (Table 1a). The mean size of larvae was of 11.7±2.9 mm (n = 960).

#### 2007 – South shore

A relatively large portion of the variation in the larval abundance data was explained by spatial variables at all the three spatial scales (Table 1b). At broad scale, nearly one third of the explained variation (32.2%) was explained by broad scale non-directional variables, whereas the directional fraction was zero (Table 1b). Therefore, the pattern at this scale was considered completely non-directional. In contrast, at medium scale, we found that both the directional and the non-directional model explained independently a significant portion of the variation (9.9 and 8.0%, respectively; Table 1b), with the total non-directional part (i.e. the pure non directional and the confounded part) accounting for 15.2% of the variation. A similar picture was found at the fine scale, but in this case, only the non-directional component was significant after partitioning (Table 1b): this latter fraction explained 13.4% of the variation in the larvae data. The mean size of larvae was of 12.6±2.2 mm (n = 1335).

### Spatial vs. Environmental Factors

#### 2005 – North shore

**Figure 2:**
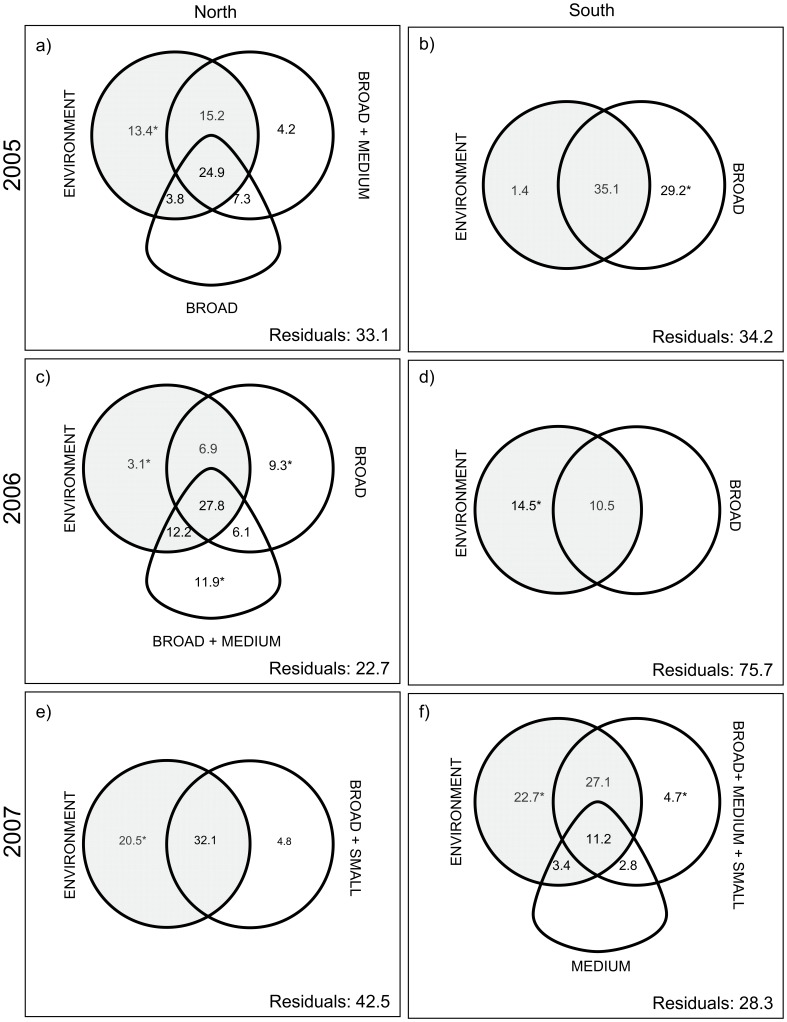
Relative importance of environmental and spatial variables. Venn diagrams illustrating the results of variation partitioning on the log-transformed abundance of yellow perch larvae per square meter in the three study years among environmental and spatial variables. The variation explained by environmental and non-directional spatial variables (i.e. MEM and TREND) is represented by circles (grey and open, respectively), whereas plectrum-shaped objects represent the variation explained by directional spatial variables (AEM). Percentage of unexplained variation is given at the bottom right of each panel (Residuals). The sizes of the objects are not proportional to their importance in terms of explained variation.

The density of emergent vegetation was the only environmental variable retained in 2005. It explained more than half of the variation in larvae abundance (Table 2a; Online Resource B: Fig. B1b). The partial contribution of the spatial component was not significant after variation partitioning and therefore it is not necessary to control for its effect in the analysis of the larvae-environment relationship. The partial relationship with larvae abundance remained significant after partialling out the effect of the spatial variables. It explained alone 13.9% of the variation in the response data (Fig. 2a; Online Resource B: Fig. B1d). The coefficient relating the density of emergent vegetation to the abundance of larvae remained significant and did not change its sign after controlling for spatial structure (Table 2a).

**Table 2:** Results for the regression models.

a)	North shore	Full model				Partial model			
**Year**	**Selected variables**	**P model**	**P**	***R*** **^2^**	**Coeff**	**P model**	**P**	***R*** **^2^**	**Coeff**
2005	**Emergent vegetation density**	<0.001	<0.001	0.573	0.252	<0.001	<0.001	0.139	0.193
2006	Day of the year	<0.001	<0.001	0.238	−0.023	<0.001	0.650	0.001	−0.006
	**Submerged vegetation structure**		<0.001	0.078	0.100		0.014	0.017	0.055
	**Emergent vegetation density**		<0.001	0.162	0.131		0.049	0.010	0.064
	Submerged vegetation density		0.004	0.046	−0.090		0.777	0.005	−0.011
2007	**Emergent vegetation density**	<0.001	<0.001	0.377	0.243	<0.001	<0.001	0.146	0.214
	Water level		0.005	0.042	−0.238		0.759	0.004	−0.046
	**Emergent vegetation structure**		0.009	0.083	−0.064		0.035	0.026	−0.056
	Conductivity		0.010	0.045	0.001		0.147	0.005	0.001
**b)**	**South shore**	**Full model**				**Partial model**			
**Year**	**Selected variables**	**P model**	**P**	***R*** **^2^**	**Coeff**	**P model**	**P**	***R*** **^2^**	**Coeff**
2005	Water level	<0.001	0.200	0.093	−0.960	0.097	n.d.	0.004	1.252
	Conductivity		<0.001	0.132	0.003		n.d.	0.001	0.001
	Day of the year		0.002	0.119	−0.125		n.d.	0.002	0.039
	Emergent vegetation structure		0.011	0.030	0.160		n.d.	0.023	0.108
	Turbidity		0.043	0.027	0.028		n.d.	0.003	−0.014
2006	**Submerged vegetation density**	<0.001	<0.001	0.132	0.055	<0.001	0.010	0.053	0.049
	**Isobath**		0.002	0.060	−0.115		0.007	0.048	−0.108
	**Emergent vegetation structure**		0.012	0.086	−0.044		0.013	0.071	−0.044
2007	Emergent vegetation density	<0.001	0.053	0.186	0.058	<0.001	0.159	0.055	0.049
	**Conductivity**		<0.001	0.235	0.003		<0.001	0.132	0.003
	Day of the year		<0.001	0.077	−0.027		0.316	0.016	−0.010
	Submerged vegetation density		<0.001	0.161	0.109		0.137	0.024	0.052

Results for the regression model relating the abundance of yellow perch larvae to environmental variables. P model = P-value of the global test of significance; P = P-value for the test of significance of a variable with all the other terms included in the model; *R* ^2^ = explained variation due to the explanatory variable (calculated as the average semi-partial *R* ^2^); Coeff = regression coefficient. Bold indicates those variables significant at the 0.05 level after controlling for spatial correlation; n.d. = not determined since the global test was not significant.

Note: The selected variables are ranked following their entry order in the full model (i.e. the model without covariables) during the forward selection procedure. Partial model: model with spatial covariables. Note that forward selection was only applied to build the full model.

The pure spatial components did not explain significant portions of the variation in larvae abundance after variation partitioning (Fig. 2a). Therefore, the spatial component (both directional and non –directional) was entirely related to the spatially-structured portion of the environmental variation (i.e. confounded between the environmental and the spatial components).

#### 2005 – South shore

In 2005, five environmental variables (water level, conductivity, day of the year, emergent vegetation structure and turbidity) were significantly related to larvae abundance (Table 2b). However, their individual contributions were relatively small and not significant after taking spatial structure into account. This reveals that the variation in larval abundance data was explained mostly by spatially structured environmental variables (Table 2b, Fig. 2b; Online Resource B: Fig. B1bcd).

Variation partitioning also showed that the response had a significant pure non-directional spatial structure at broad scale (29.2%) (Fig. 2b).

#### 2006 – North shore

In 2006, four environmental variables (day of the year, submerged vegetation structure, emergent vegetation density and submerged vegetation density) were significantly related to larvae abundance (Table 2a). However, only submerged vegetation structure and emergent vegetation density were significant after controlling for spatial correlation. The variable describing the structure of submerged vegetation was significantly related to larvae abundance and seemed to be related more to the presence-absence of submerged vegetation than to its structure: based on the PCA scores for the qualitative variables, sites with either linear or arbustive vegetation had in fact higher abundances of larvae than sites without submerged vegetation (results not shown). The density of emergent vegetation was positively related to larvae abundance, and remained significant after partialling out the effects of spatial structure (Table 2a), albeit it explained a smaller fraction of the variation than the previous year.

Among the spatial variables retained, both directional (broad and medium-scale) and non-directional (broad) variables were significantly related to larvae abundance after variation partitioning; they explained alone 11.9% and 9.3% of the variation in the response, respectively (Fig. 2c). More than 45% of the spatial variation was confounded with the environmental variables (Fig. 2c; Online Resource B : Fig. B2bcd).

#### 2006 – South shore

All three selected environmental variables, the abundance of submerged vegetation, the isobaths and the structure of emergent vegetation, were significantly related to larvae abundance after controlling for spatial correlation (Table 2b). The abundance of submerged vegetation was associated to higher larvae abundances whereas the isobaths was inversely related to it. Emergent arbustive vegetation was associated to higher larvae abundances than vegetation with a linear structure. Overall, the pure environmental component explained 14.7% of the variation in the larval abundance data (Online Resource B: Fig. B2d). All broad-scale non-directional variation was confounded with the environmental variables (Fig. 2d).

#### 2007 – North shore

As in 2005, a large portion of the variation (37.7%) in larvae abundance was directly related to the density of the emergent vegetation on the north shore (Table 2a). The structure of emergent vegetation was also significantly related to the abundance of larvae, but explained a smaller fraction of the variation (8.3%). In contrast to the south shore in 2005, this variable suggests that larvae were more associated to the linear than to the arbustive vegetation structure. All these variables remained significantly related to the abundance of larvae and did not change the sign of their coefficient after taking spatial structure into account (Table 2a). In contrast, conductivity and water level were related to larvae abundance only before taking spatial structure into account. Variation partitioning showed that the environmental variables were partly spatially structured (32.1%), but a relatively large portion of their variation was significantly related to larvae abundances after controlling for spatial structure (20.5%) (Online Resource B: Fig. B3bcd). The environmental variables were structured at both broad and small scales and had a non-directional spatial structure. Virtually no pure spatial variation remained after variation partitioning (Fig. 2e; Online Resource B : Fig. B3c); all the variation explained by small- and broad-scale non-directional spatial variables was confounded with the environmental variables.

#### 2007 – South shore

In 2007, the environmental variables explained together a large fraction (64.5%) of the variation in the larval abundance data. More than half of this variation was spatially structured (Fig. 2f, Online Resource B: Fig. B1bcd) but only the non-directional spatial component was significant after variation partitioning. If the non-significant directional part is not taken into account, the pure spatial and environmental components, both significant, represented respectively 4.7 and 22.7% of the variation in the larval abundance data. The pure environmental fraction was mostly related to conductivity, which was directly related to the abundance of larvae (Table 2b; Fig. 2f).

## Discussion

The distribution of yellow perch larvae in LSP revealed in several occasions both directional and non-directional patterns, depending on the spatial scale considered. However, only in one case did the pure directional component explain a significant fraction of the variation in larval abundance (i.e. the north shore in 2006, Fig. 2c). Moreover, most of the spatial patterns of larvae were shared with the environmental variables. Notwithstanding the confounding between directional and non-directional forces, our results suggest at best a moderate role for downstream dispersal of yellow perch larvae in this system. Furthermore, a positive association between larvae and environmental variables (mostly aquatic vegetation), independent of spatial structure, suggests that habitat selection is a factor determining yellow perch larvae distributions. Taken together, our results suggest that yellow perch larvae cannot be considered merely as passive particles in LSP. By explicitly analyzing the relative importance of directional processes at different scales, the statistical approach used here helped us extract this crucial information from the larval abundance data.

As predicted, broad-scale non-directional patterns were detected on both shores for each study year, confirming our prediction (i). We interpret these patterns as being related to the locations of the main spawning grounds (Fig. 1, Online Resource B). In LSP, spawning yellow perch aggregate in the main bays, which are densely vegetated and in the spring offer extensive spawning grounds in shallow flooded areas [8]. The higher vegetation abundance in the bays relatively to the rest of the lake probably explains why the larvae distribution has a strong non-directional component, resulting from the overlapping of spawning grounds with spatially structured environmental factors. The absence of directionality in most broad-scale patterns is not surprising since, according to our prediction (ii), we did not expect current to strongly affect larvae distribution at this spatial scale along the sampled isobaths. However, it is difficult to know if the swimming speed of yellow perch larvae was sufficient for larvae to avoid drift at the selected isobaths during our sampling. The available hydrodynamics data in LSP in other years suggest that water velocities at the sampled isobaths are relatively slow (<10 cm sec^−1^) [21], but within a range in which drift cannot be excluded. Only in one case (north shore in 2006, Fig. 2c), we detected a significant pure directional pattern, at both broad and medium scales, suggesting that in some situations drift is a driving force. This finding suggests that in our system directional processes can affect the distribution of larvae, probably because these latter occur at the edge of backwaters and at current speed where larvae are not capable of swimming for long periods. However, their relatively low occurrence suggests that drift is a minor component of early larvae dispersal in LSP, at least at the sizes observed here, giving only weak support to our second prediction. The lack of any other pure directional pattern at medium and small scales corroborates this point. *Perca* spp. larvae are known to move out into the pelagic area and after some time return to shallow-water habitats [5], [34], [35]. There is a lack of agreement, however, as to how (i.e. passive vs. active), when and why these changes take place (e.g. see [7]). *Perca* spp. appear to be adapted to variable environments and, depending on the system, the initiation of post-hatching dispersal has been considered as merely passive [3] or more active [6]. Our results give indirect support to the hypothesis that the newly-hatched larvae could make an active habitat choice, at least in the absence of strong currents [6], [36]. Currents probably play a greater role in the dispersal process in some systems, as in the Canadian Great Lakes where currents are relatively strong (10–20 cm·s^–1^) and yellow perch larvae can be found more than 50 km offshore [3], [4]. Our results give low support to the passive advection hypothesis, by showing that directional processes have a small importance in our atypical study system, which is characterized by vast macrophyte beds. This is not enough to exclude a role for passive transport due for example to wind-induced currents or changes in water level, especially in deeper offshore areas not sampled in our study. However, the consistent relationship with the density of emergent vegetation after controlling for spatial structure suggests that larvae can select their habitat, at least at the time of our sampling (i.e. 2 weeks ca. after hatching). This might be similar to what has been found for the larvae of marine reef fishes, previously considered mainly as passive particles transported by currents, and which are now considered to be selecting where they settle [37]. To our knowledge, potential active habitat selection by freshwater fish larvae has been the object of very few studies to date. By combining two statistical methods currently available, our approach suggests a simple and new way to analyze the relative importance of different hypothesis about the role of passive transport versus active swim to settlement habitats.

The association of larvae with vegetation may also be partly explained by three other hypotheses not associated with habitat choice: i) a lack of horizontal offshore migration, (ii) a “sediment trap” effect in macrophyte beds, and (iii) a reduction of larval mortality in macrophyte beds. Hypothesis (i) is in contrast with the current paradigm of yellow perch larvae leaving the littoral vegetated areas just after hatching [4], [5], [7]. The absence of a typical deep pelagic zone in LSP would possibly contribute to this pattern. However, this is only partly in agreement with our personal observations in LSP, since egg strands are typically found on the temporarily flooded areas, relatively far from our sampling sites. Moreover, the association with the vegetation remained significant after controlling for the effect of the spatial locations of the bays, suggesting that the observed relationship is at least partly independent of bay locations. Hypothesis (ii) suggests that since macrophyte beds reduce locally current velocity, they might trap drifted fish larvae as these were sediment particles. We cannot exclude this hypothesis, but this seems at odds with the results of the AEM analysis, which showed only little evidence of directional processes. However, it is also possible that, by counteracting the effects of drift, this mechanism would reduce artificially the directional component of the model. In contrast, it is possible that the reduced current speed within macrophyte beds is a condition sought by larvae, in accordance with the habitat choice hypothesis. Hypothesis (iii) suggests that larvae mortality (e.g. predation-dependent) is higher in open water areas and that the observed patterns might be simply due to the higher survival into macrophyte beds. Current knowledge is inadequate to address this controversial hypothesis (e.g. [7]) and studies explicitly design to test this hypothesis would be needed to answer this question.

Weak or absent downstream drift combined with habitat selection by larvae might reduce gene flow within the lake. For *P*. *fluviatilis*, it has been shown that genetic differentiation is possible not only in large [38], [39] but also in small lakes in the absence of evident barriers to gene flow [40]. Genetic analyses on a large number of AFLP genetic markers in larvae from LSP in 2004 suggest that genetic differences exist among individuals from the fours known spawning grounds (Fig. 1), even on the same shore of the lake [41]. The limited larval drift hypothesis suggested by our results is in accordance with this finding.

In general, the directional spatial variables tended to model the larval abundance data more efficiently than the non-directional ones since they modelled appropriately broad-scale patterns without previous detrending and using relatively few eigen vectors to model the same spatial structures (results not shown). However, our results indicate that this is not enough to infer the presence of a directional pattern in the data, since directional spatial variables in most cases did not extract more information from the larval abundance data than non-directional ones. Therefore, our results also show that AEM analysis alone cannot be used to infer the importance of directional patterns, and that only the comparison with a non-directional counterpart (e.g. MEM) can help to reach this goal. This approach may help in revealing the potential for broad-scale physical transport processes when only the patterns of larval distribution are known. We hope that our results will encourage future research using this approach in other systems, to assess the relative importance of directional and non-directional processes in determining the distribution of living organisms.

By revealing a strong link between larvae and the structure of the habitat, our results suggest that settlement of yellow perch larvae could be predicted from simple habitat variables. A better understanding of the habitat-larvae relationship will improve our ability to predict recruitment success in fish and help in planning better management strategies to protect their populations.
